# FEASIBILITY OF AN ONLINE DEMENTIA CAREGIVER EDUCATION CURRICULUM FOR FAITH-COMMUNITY LEADERS

**DOI:** 10.1093/geroni/igad104.3353

**Published:** 2023-12-21

**Authors:** Jeffrey Graupner, Katherine Thompson, Monica Long, Tiffany Smith, Jenil Bennett, Shellie Williams

**Affiliations:** University of Chicago, Chicago, Illinois, United States; University of Chicago, Chicago, Illinois, United States; University of Chicago/Share Network, Chicago, Illinois, United States; University of Chicago/Share Network, Chicago, Illinois, United States; University of Chicago, Chicago, Illinois, United States; University of Chicago, Chicago, Illinois, United States

## Abstract

Dementia caregivers have well-documented needs including dementia education, resources, and emotional support. Our prior work demonstrated the efficacy of a novel dementia education program designed to transform urban faith-community leaders into “Dementia Resource Champions” equipped to start their own support groups. We adapted this program to be delivered virtually. A 6-week curriculum of presentations, resources, and video interviews with caregivers was created by an interprofessional team of geriatrics specialists. Recruitment for “DRC: Online” utilized email blasts and online sign-up forms. While registration for the course was open to all, recruitment emphasized that this program was originally developed for local faith-based communities. Each week, participants received an email with a pre-recorded video didactic presentation, a caregiver interview video, and a link for the weekly videoconference discussion. Fourteen participants completed the pre-training survey, and seven went on to complete the 6-week course and post-training survey. Participants’ reported self-confidence in dementia knowledge increased significantly from pre- to post-training (5-Likert Scale, Paired t-test: Pre (M = 2.1 , SD = 1.1); Post (M = 4, SD = 0.6), t(6) = 7.1, p < .001). Self-reported ability to use the internet to find dementia resources also increased significantly (Pre: (M = 3.9, SD = 1.1); Post: (M = 4.7, SD = 0.5), t(6) = 2.5, p = .045.) Participants’ average rating of the program was 4.7 (5-Point Likert, 5=Excellent). Results indicate that this relatively low-tech approach to virtual dementia education is a feasible way to train community leaders to become resources for dementia caregivers.

